# Intrapericardial Administration of Mesenchymal Stem Cells in a Large Animal Model: A Bio-Distribution Analysis

**DOI:** 10.1371/journal.pone.0122377

**Published:** 2015-03-27

**Authors:** Rebeca Blázquez, Francisco Miguel Sánchez-Margallo, Verónica Crisóstomo, Claudia Báez, Juan Maestre, Mónica García-Lindo, Alejandra Usón, Verónica Álvarez, Javier G. Casado

**Affiliations:** 1 Stem Cell Therapy Unit, Minimally Invasive Surgery Centre, Caceres, Spain; 2 Endoluminal Therapy and Diagnosis, Minimally Invasive Surgery Centre, Caceres, Spain; 3 Anaesthetics Unit, Minimally Invasive Surgery Centre, Caceres, Spain; Indiana University School of Medicine, UNITED STATES

## Abstract

The appropriate administration route for cardiovascular cell therapy is essential to ensure the viability, proliferative potential, homing capacity and implantation of transferred cells. At the present, the intrapericardial administration of pharmacological agents is considered an efficient method for the treatment of cardiovascular diseases. However, only a few reports have addressed the question whether the intrapericardial delivery of Mesenchymal Stem Cells (MSCs) could be an optimal administration route. This work firstly aimed to analyze the pericardial fluid as a cell-delivery vehicle. Moreover, the *in vivo* biodistribution pattern of intrapericardially administered MSCs was evaluated in a clinically relevant large animal model. Our *in vitro* results firstly showed that, MSCs viability, proliferative behavior and phenotypic profile were unaffected by exposure to pericardial fluid. Secondly, *in vivo* cell tracking by magnetic resonance imaging, histological examination and Y-chromosome amplification clearly demonstrated the presence of MSCs in pericardium, ventricles (left and right) and atrium (left and right) when MSCs were administered into the pericardial space. In conclusion, here we demonstrate that pericardial fluid is a suitable vehicle for MSCs and intrapericardial route provides an optimal retention and implantation of MSCs.

## Introduction

Clinical and preclinical studies have shown that multipotent stem cells can be successfully used for the improvement of cardiac function [1–3]. Although there are quite a few stem cell products in the market [4], many different clinical trials are continuously demonstrating that MSCs are a promising cell source for regenerative therapy [5,6]. These cells fulfill the safety requirements being particularly attractive for their availability, multipotentiality, self-renewal ability and low immunogenicity [1,7,8]. The appropriate route for MSCs administration is a fundamental step for the success of stem cell-based therapies and determines their therapeutic effect. At the present, there are many clinical trials being conducted using different administration routes. Some of the most common administration routes for cell delivery are: direct surgical intramyocardial injection, catheter-based intramyocardial administration (transcoronary venous or transendocardial approach), intravenous infusion, intracoronary artery administration or retrograde coronary venous delivery [9].

Several pros and cons are attributed to any of these routes. For example, intracoronary administration produces a more uniformly distributed pattern of MSCs [1] but may result in blockage of coronary arteries [10,11]. The intramyocardial delivery appears to have a higher retention rate although there is a significant loss of transplanted cells due to myocardial contraction [12]. Intravenous infusion is the easiest method for cell delivery but its retention rate is very low [13]. At the present, most of the preclinical studies have clearly demonstrated that the retention of transplanted cells in the heart is very low by any delivery method [14].

Although there are still so many other open questions that need to be answered (dose, timing or cell type), alternative techniques and administration routes need to be investigated to ensure the viability of transferred cells, proliferative/differentiation potential as well as their homing capacity. Moreover, it would be advisable to guarantee the implantation of cells for a period of time enough to reach the desired therapeutic effect. In this sense, a higher retention rate may have a greater impact on cardiac repair enabling paracrine stimulation through the release of growth factors, pro-angiogenic molecules, immunomodulatory factors, proliferative and anti-apoptotic molecules.

Several administration routes are currently being studied for clinical use [12], but only a few reports address the question whether the intrapericardial delivery of MSCs could be a safe and effective alternative to other surgical procedures. The pericardial fluid (PF) is an ultrafiltrate of plasma secreted by the serous membrane to decrease the friction between heart and adjacent tissues. The composition is very similar to plasma (with lower concentrations of proteins, triglycerides and cholesterol) and could be somehow considered an optimal vehicle to preserve the viability and functionality of MSCs.

Compared to other routes, the pericardial delivery permitted the administration of very high doses. On the contrary, the intramyocardial delivery is limited by volume and several adverse effects (i. e. arrhythmias) have been described [15,16]. In the case of intravenous and intracoronary routes, the main disadvantage is the low retention rate in the heart with a significant number of MSCs trapped in the lungs [17]. In contrast, PF provides a low turnover rate, allowing a long-term persistence of transferred cells. Additionally, it is important to note that, in the case of intracoronary administration of MSCs, these cells may induce a myocardial damage by microvascular obstruction [18,19], which is not a problem when injected intrapericardially, since this route is independent of impaired vascular functions which lead to myocardial infarction.

At the present, preclinical studies have shown that intrapericardial administration is an efficient method for delivering pharmacological agents [20,21]. Preclinical tests in large animal models have been performed using Fibroblast Growth Factor, L-Arginine or omega-3 fatty acids [22–24]. The treatment with Fibroblast Growth Factor in a porcine model of chronic myocardial ischemia has demonstrated a beneficial effect increasing the myocardial vascularity without adverse effects [25]. The delivery of L-Arginine has been tested in dogs reducing the severity of ischemic ventricular arrhythmias [23]. Finally, the intrapericardial delivery of omega-3 fatty acids reduced malignant arrhythmias and infarct sizes in a porcine infarct model [24].

In clinical settings, the blood supply to the myocardium becomes insufficient to those patients with arterial obstructions. This obstruction might also compromise the delivery of transferred cells. The PF is independent of the vascular niche, has a low turnover rate that may provide a long term effect to achieve the desired therapeutic effect of stem cells.

Here we hypothesize that intrapericardial administration of MSCs may retain the cells in close proximity to the injury preserving its viability, homing and bioactivity. We also hypothesize that, the presence of MSCs in the PF, through the release of paracrine factors, may have a beneficial effect providing an optimal microenvironment for promoting cardiac repair.

In this work, our *in vitro* experiments have demonstrated for the first time that, PF preserves the phenotype, survival and proliferation rates of MSCs. The iron oxide labeling permitted *in vivo* tracking of intrapericardially administered MSCs by magnetic resonance imaging (MRI) in a clinically relevant animal model. The engraftment of MSCs in pericardium, ventricles and atrium was confirmed by histological examination and PCR detection. Although new medical devices such as AttachLifter has been developed to provide a secure implantation of cells in the pericardial space [26], to our knowledge, this is the first *in vitro* and *in vivo* imaging study where the distribution of MSCs after intrapericardial administration is finely detected. Altogether, our results suggest that PF could be considered an optimal vehicle for MSCs and intrapericardial administration an advantageous route for cardiovascular cell therapy.

## Materials and Methods

### Isolation of porcine bone marrow-derived mesenchymal stem cells

All experimental protocols were approved by the Committee on the Ethics of Animal Experiments of Minimally Invasive Surgery Centre and fully complied with recommendations outlined by the local government. All surgery was performed under sevofluorane anesthesia, and all efforts were made to minimize suffering. Allogeneic bone marrow-derived mesenchymal stem cells (pBM-MSCs) were isolated from femurs of Large White male pigs aged between 3–4 months and weighed between 25–30 kilograms. The cell suspension was filtered through a 40 μm nylon mesh and mononuclear cells were isolated by centrifugation over Histopaque-1077 (Sigma, St. Louis, MO, USA). Mononuclear cells were recovered and washed twice with PBS. These cells were resuspended in DMEM containing 10% fetal bovine serum (FBS), seeded onto tissue culture flasks and expanded at 37°C and 5% CO_2_. Following 48 hours in culture, the non-adherent hematopoietic cells were removed. Adhered cells were passaged at 80%–90% confluence by 0.25% trypsin solution (Lonza, Walkersville, Inc.) and seeded to a new culture at a density of 5000–6000 cells/cm^2^. Culture medium was changed every 7 days. The pBM-MSCs at passages 10 to 15 were used for intrapericardial delivery.

### Biochemical analysis of pericardial fluid and plasma

Before intrapericardial injection of pBM-MSCs, blood and PF samples from animals were collected, centrifuged to eliminate cellular debris and processed in the random access clinical analyzer Metrolab 330 (Metrolab S.A., Buenos Aires, Argentina) to determine their biochemical composition (total bilirubin, calcium, cholesterol, creatinine, glucose, phosphorus, total proteins, triglycerides and urea concentrations). The measurements were compared by the Student's *t* test and paired test using SPSS software. A *p* value ≤ 0.05 was considered significant.

### CCK-8 proliferation and viability assays

The CCK-8 method is a cell viability assay which measures the activity of living cells by assessing their mitochondrial activity. In order to quantify the proliferative activity of pBM-MSCs, these cells were co-cultured in the presence of PF. The PF was aspirated from three euthanized healthy animals, centrifuged for 5 min at 450 x g and passed through a 0.22 μm filter to remove cell debris. The pBM-MSCs were seeded in DMEM medium (without phenol-red) at a density of 5000 cells/cm^2^ in 96 well plates. Different concentrations of PF were prepared (12.5%, 25%, 50%, 75% and 100%). The CCK-8 (Sigma, St. Louis, MO, USA) was added according to the manufacturer's protocol and absorbance was read at 450 nm. The viability was measured by trypan blue dye-exclusion assay. Data were statistically analyzed with one-way analysis of variance (ANOVA) using SPSS-15 (SPSS, Chicago, IL, USA).

### Phenotypic analysis of mesenchymal stem cells by flow cytometry

For flow cytometric analysis, the pBM-MSCs were cultured in the presence of PF at different concentrations (12.5%, 25%, 50%, 75% and 100%) and under standard culture conditions (10% FBS). At day 7 and 14, the cells were detached with 0.25% trypsin solution and stained with FITC-conjugated human monoclonal antibodies (mAbs) against CD90, CD105 (both cross-reactive with porcine) and porcine mAbs against CD29, CD31, CD44, CD45 and Swine Leukocyte Antigens (Class I and Class II) from Serotec (Kidlington, UK). The phenotypic analysis was performed as follows: 2x10^5^ cells were incubated for 30 min at 4°C with appropriate concentrations of mAbs in the presence of PBS containing 2% FBS. The cells were washed and resuspended in PBS. The flow cytometric analysis was performed on a FACScalibur cytometer (BD Biosciences, San Jose, CA, USA) after acquisition of 10^5^ events. Cells were primarily selected using forward and side scatter characteristics and fluorescence was analyzed using CellQuest software (BD Biosciences, San Jose, CA, USA). Isotype-matched negative control antibodies were used in all the experiments. The mean relative fluorescence intensity (MRFI) was calculated by dividing the mean fluorescent intensity (MFI) by the MFI of its negative control. Data were statistically analyzed with one-way analysis of variance (ANOVA) using SPSS-15 (SPSS, Chicago, IL, USA).

### *In vitro* assessment of superparamagnetic iron oxide labeling in pBM-MSCs

The superparamagnetic iron oxide (SPIO) nanoparticles offer an optimal signal for T2 weighted magnetic resonance images acting as contrast agents in MRI. For this, in order to improve the *in vivo* cell tracking of pBM-MSCs, these cells were magnetically labeled with increasing SPIO concentrations (Endorem, Guerbet, Paris, France). The SPIO solution was previously coated with Poly-L-Lysine (Sigma, St. Louis, MO, USA) at 0.02 mg/mL (MW = 389,000 Daltons) as a facilitator agent. Both SPIO and Poly-L-Lysine were gently shaken for 30 minutes at room temperature and added to adherent cell cultures at a proportion of 1:1 in DMEM containing 10% FBS. The final concentrations in all groups were 25, 50, 100 and 200 μg/mL of SPIO. The labeling efficiency was *in vitro* determined by Prussian Blue staining.

### Superparamagnetic iron oxide detection and fluorescent labeling

After incubation with SPIO, the Prussian Blue method was used to detect SPIO particles within the cells. The *in vitro* cultured pBM-MSCs were fixed in 4% paraformaldehyde at 37°C for 30 min. After fixation, the samples were washed with PBS and incubated with equal volume of 8% hydrochloric acid and 4% potassium ferrocyanide for 20 min at room temperature. Cultures were washed with distilled water and visualized after 24h, 3d, 5d and 7d by light microscopy. The intracellular content of potassium ferrocyanide was spectrophotometrically quantified. *In vitro* cultured cells were lysed in the presence of 0.1% Triton X-100 (Sigma, St. Louis, MO, USA) and the absorbance of the extracts was quantified at 700 nm. For the fluorescent labeling of cells, the Vybrant CM-DiI cell labelling solution (Invitrogen, Carlsbad, CA) was added to normal culture media according to manufacturer's recommendations, to uniformly label cells.

### Myocardial infarction model creation

Four Large White pigs were housed in the animal facility at Minimally Invasive Surgery Centre and used for all experimental procedures. Animals were aged between 3–4 months and weighed between 30–35 kilograms. Animal care and all experimental procedures were approved by the Ethics Committee for Animal Research of the local government. Each animal was premedicated with diazepam 0.1 mg/kg, ketamine 10 mg/kg, and atropine 0.01 mg/kg intramuscularly. Intravenous hydration with normal saline was established by catheterization of the auricular vein with 18–22 gauge needles (Abbott Ireland) and maintained during procedures. Induction of anesthesia was performed intravenously with 2 mg/kg of propofol. After the pig was endotracheally intubated, it was connected to a system for anesthesia (Ohmeda Excel 210) and a mechanical ventilator Ohmeda 7800 (Ohmeda, Madison, Wis). Anesthesia was maintained with 2.0%–2.5% halothane, and blood pressure, electrocardiogram, O_2_ saturation and end tidal CO_2_ were monitored closely throughout the procedure. The pigs were fixed on the operating table in the supine position with cranial and caudal extension of the limbs. The thorax and upper abdomen were shaved and draped in a sterile fashion. Continuous infusion of lidocaine at rate of 1 mg/kg/h (Lidocaina Braun, Braun Medical, Barcelona, Spain) was used through the procedure. Systemic heparin (Heparina Rovi 5%, Laboratorios farmaceuticos Rovi, Madrid, Spain) was injected intravenously (150 UI/Kg) prior to percutaneous sheath placement. Under aseptic conditions, a right femoral arterial access was established using the Seldinger technique and a 7 Fr introducer sheat (Terumo, Tokyo, Japan) was placed percutaneously into the femoral artery. Under fluoroscopic guidance (Philips Mobile Digital Angiographic System-BV Pulsera, Philips Medical System, Best, The Netherlands), a 6 Fr hockey stick guiding catheter (Mach 1, Boston Scientific Corporation, Natick, MA, USA) was introduced and placed at the origin of the left coronary artery. Coronary angiograms were obtained in the 40° left anterior oblique projection to better demonstrate the length of the Left Anterior Descending artery (LAD), and a 0.0014 coronary guidewire (Hi-torque, Abbott Vascular, Santa Clara, CA, USA) was advanced inside the LAD. After measuring the diameter of the LAD immediately below the origin of the first diagonal, an over-the-wire PTCA balloon of appropriate diameter (typically 3mm, Apex OTW, Boston Scientific, Natick, MA, USA) was advanced to this location and inflated to occlude the LAD flow for 90 min. A lidocaine bolus was also administered immediately before balloon inflation and deflation. Upon balloon deflation, the coronary artery was checked for patency by repeating angiogram. Animals were maintained fully monitored under general anesthesia for 45 min after infarct induction, in order to treat any malignant arrhythmias that may ensue.

Cardiac MRI was performed before the creation of the model and 7 days post-myocardial infarction using a 1.5 T MR system (Intera 1.5T Philips Medical System, Best, The Netherlands). For *in vivo* cell tracking, cardiac MRI images were obtained at different time points for one week. All imaging was performed under general anesthesia using retrospective cardiac gating with the animal in sterna decubitus and a four elements phase array coil was placed around the animal chest. Images were acquired in the intrinsic cardiac planes: short axis, long axis and four chamber views. For measurement of ventricular function and mass breath hold balanced SSFP, cine images were obtained over the entire ventricle. For infarct size measurements, images were acquired 5–15 min after the injection of 0.2 mmol/kg of a gadolinium-based contrast agent using a breath hold 3D gradient-echo inversion-recovery sequence. MR images were analyzed for a left ventricular volume, mass, function and infarct size.

### Intrapericardial administration of pBM-MSCs in non-infarcted and infarcted porcine hearts

Each animal was pre-medicated with diazepam 0.3 mg/kg and ketamine 10 mg/kg intramuscularly. Intravenous hydration with normal saline was established by catheterization of the auricular vein with 18–20 gauge needles (Abbott, Sligo, Ireland) and maintained during procedures. Induction of anesthesia was performed intravenously with 2 mg/kg of propofol. After the pig was endotracheally intubated, it was connected to a system for anesthesia (Leon Plus, Heinen+Löwenstein). Anesthesia was maintained with 1.8%–2% sevofluorane, and blood pressure, electrocardiogram, O_2_ saturation and end tidal CO_2_ were monitored closely throughout the procedure. The pigs were fixed on the operating table and thorax and upper abdomen were shaved and draped in a sterile fashion. Intrapericardial injections containing 100 x 10^6^ pBM-MSCs in 5 mL of Hypothermosol (BioLife Solutions, Inc., Owego, NY) were performed via thoracotomy using an Abbocath-T 20G catheter (Hospira, Lake Forest, IL). Once the injections were completed, the incision was closed in layers and the animals were allowed to recover. The animals were euthanized 7 days later with a lethal dose of Potassium Chloride intravenously infused under general anesthesia. The hearts were firstly examined *in situ*. Gross visual inspection was focused on possible complications associated with the procedures and potential damages to the pericardium, epicardium and surrounding structures in the mediastinum.

### *In vivo* visualization of intrapericardially delivered pBM-MSCs by magnetic resonance imaging

The pBM-MSCs were labeled with 100 μg/mL of SPIO for 24h as previously described. A total of 100 x 10^6^ labeled pBM-MSCs cells were injected into the pericardial cavity (see previous section in Materials and Methods). To perform MRI, the animals were anesthetized with isofluorane (2–3% in medical air administered via a nose cone). Cardiac-MRI was performed using a 1.5 T MR system (Intera 1.5 T, Philips Medical Systems, Best, The Netherlands). All imaging was performed using retrospective cardiac gating, with the animal in sternal decubitus position and a four elements phase array coil was placed around the animal´s chest. Images were acquired in the intrinsic cardiac planes: short axis, long axis and four chamber views. For measurements of ventricular function and mass breath hold balanced steady-state free precession, cine images were obtained over the entire ventricle. The SPIO-labeled pBM-MSCs were identified with a T2-star weighted breath hold gradient-echo sequence. The resonance images were taken before injection, immediately after the injection and at days 3, 5 and 7 post-injection.

### Tissues sampling and histology

Samples were obtained after euthanasia. Firstly, the PF was aspirated from the pericardial cavity using an Abbocath-T 20G catheter. The PF was centrifuged for 5 min at 450 x g. The pellet was frozen for subsequent DNA amplification and supernatants were passed through a 0.22 μm filter to remove cell debris for subsequent biochemical analyses. The parietal pericardium was carefully removed and samples were frozen for PCR analysis or fixed in paraformaldehyde 4% for histological analyses. The heart was carefully dissected and samples from pericardium, right and left ventricles, right and left atrium, interventricular septum and interauricular septum were frozen or fixed.

### Y chromosome PCR

For the detection of male-derived pBM-MSCs in female hearts, Y-chromosome PCR was carried out from tissue samples. DNA extraction was performed with TRI Reagent (Sigma, St. Louis, MO, USA) according to the manufacturer's instructions. The detection of male cells in a female recipient was carried out by PCR using the Taq DNA Polymerase (Invitrogen, Carlsbad, Ca). Amplification consisted in 40 cycles of 30 seconds at 94°C for melting, 30 seconds at 55°C for annealing and one min at 72°C for amplification. The primers 5´-ACAGAGGGCCTATTCATCTCAG-3´ (forward) and 5´-CTTAATGGCTAATCACGGGAAC-3´ (reverse) were designed to allow the amplification of Y-chromosome specific sequences (NCBI Reference Sequence: NC_010462.2).

### Histological examination

The animals were sacrificed at day 7 post-administration. The heart was removed, rinsed, and sliced into 1–3 cm short-axis sections for histological studies. Histological samples were fixed in 4% paraformaldehyde, paraffin-embedded and cut in 5–8μm thickness. The sections were stained for Prussian blue and eosin and histologically examined to identify the accumulation SPIO-labeled cells in myocardium and parietal pericardium. Finally, histological samples were stained for Masson Trichrome and Toluidine blue.

## Results

### Viability, proliferative behavior and phenotype of pBM-MSCs in pericardial fluid

**Fig 1:**
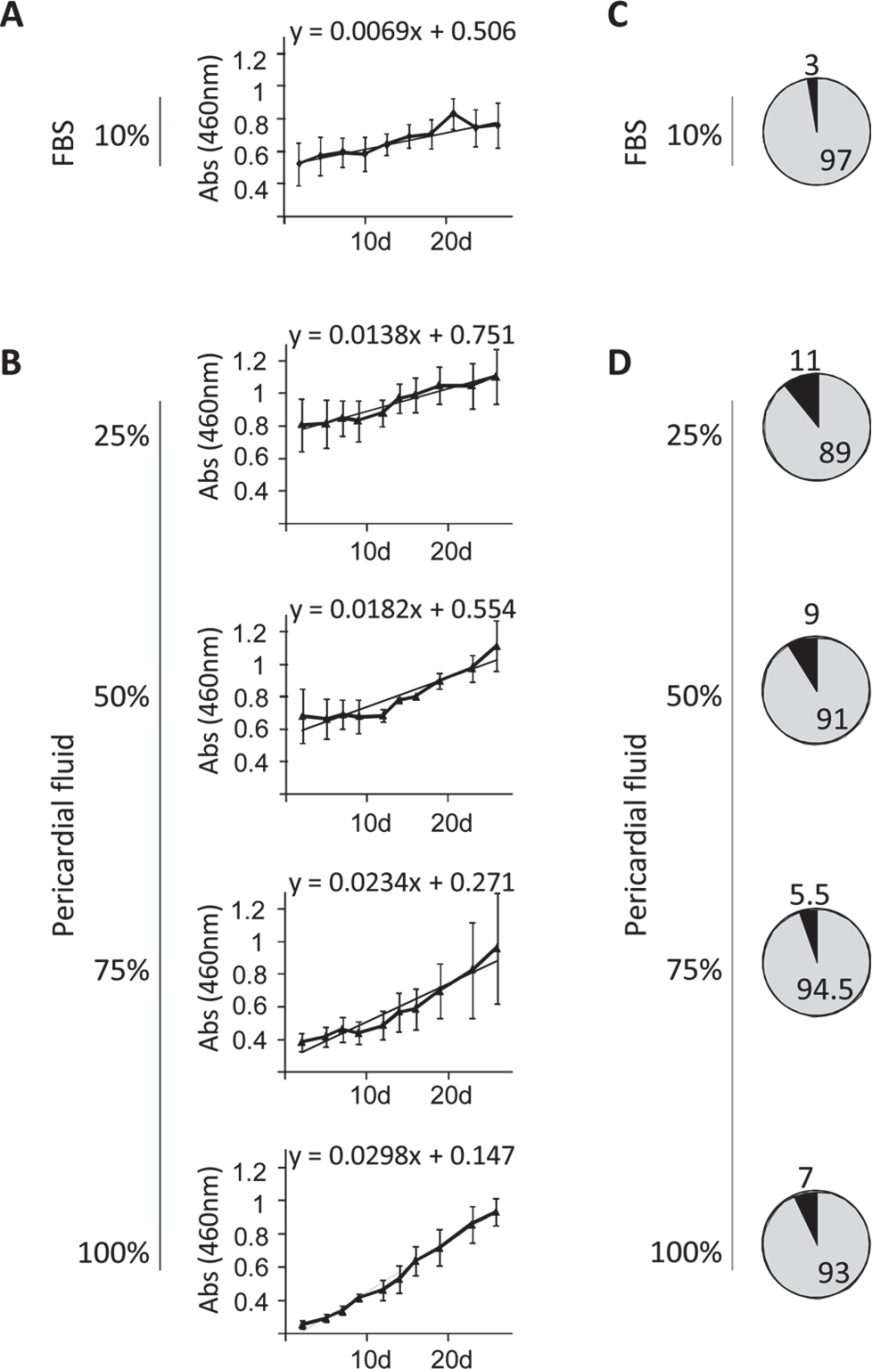
Proliferative behavior and viability and of pBM-MSCs co-cultured with pericardial fluid. The pBM-MSCs were co-cultured in the presence of pericardial fluids from three different healthy animals. (A and B) The proliferative behavior of cells was spectrophotometrically quantified at 460 nm for 26 days using a CCK-8 proliferation kit. Values shown in the graphics represent mean±SD of 3 independently performed experiments. The slopes correspond to the proliferation rates of pBM-MSCs. An ANOVA test was performed comparing the slopes of the different experiments between groups, and no significant differences were found. (C and D) The percentage of viable cells was calculated by trypan blue dye-exclusion and the average percentage of live and dead cells is represented in grey and black respectively.

Preliminary experiments were firstly conducted to compare the biochemical composition of PF with plasma. As shown in Table 1, significant differences were found for cholesterol, phosphorus, triglycerides and total proteins, however, the rest of analyzed parameters were very similar. Once compared the biochemical composition, in order to be sure that PF is an adequate vehicle for cell administration, the viability of pBM-MSCs was assayed in the presence of different concentrations of PF. Healthy animals were euthanized and their PFs were *in vitro* cultured together with pBM-MSCs. The viability and proliferative behavior were calculated by trypan blue dye-exclusion and CCK-8 proliferation assays respectively. As shown in Fig. 1, the viability was not affected at any of the tested concentrations being comparable to control cells cultured under standard conditions (DMEM together with 10% FBS). Additionally, as the composition of PF may modify the proliferative behavior of pBM-MSCs, cell proliferation assays were performed in the presence of different concentrations of PF. These proliferation experiments were conducted using Cell Counting Kit-8 (CCK-8) and a cell growth curve was generated (slopes from Fig. 1 show the proliferation rates). Our *in vitro* results clearly demonstrated that, the proliferative rate of pBM-MSCs was unaffected by the presence of PF. More importantly, when we compared the proliferative rates of pBM-MSCs cultured under standard conditions with those cells cultured in the presence of PF, we found that cell proliferation increased proportionally, although not significantly, to PF concentrations.

**Table 1:** Biochemical analysis of pericardial fluid and blood plasma (n = 3).

	**Percardial fluid**	**Blood plasma**
	**Mean ± SD**	**Mean ± SD**
**Total bilirubin (mg/dl)**	0.03 ± 0.02	0.23 ± 0.11
**Calcium (mg/dl)**	7.87 ± 2.12	11.47 ± 1.33
**Cholesterol (mg/dl)** *	4.00 ± 1.00	81.00 ± 8.66
**Creatinine (mg/dl)**	2.20 ± 0.07	2.24 ± 0.09
**Glucose (mg/dl)**	86.67 ± 3.21	82.67 ± 13.05
**Phosphorus (mg/dl)** *	5.47 ± 1.36	6.49 ± 1.03
**Total protein (g/dl)** *	0.95 ± 0.29	5.82 ± 0.65
**Triglycerides (mg/dl)** *	14.33 ± 5.51	88.67 ± 38.55
**Urea (mg/dl)**	18.03 ± 2.96	20.20 ± 2.95

*p ≤0.05 in a paired Student's t-test.

**Fig 2:**
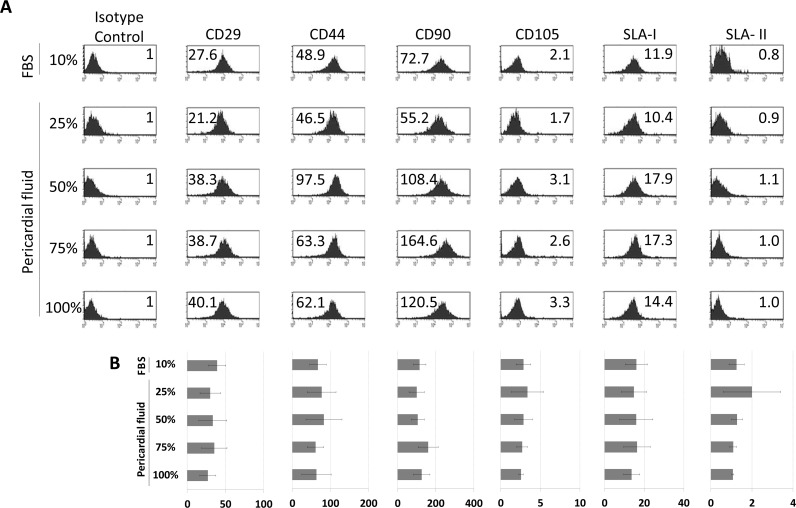
Effect of pericardial fluid on pBM-MSCs phenotype. The phenotypic analysis was performed by multicolor flow cytometry. The cells were *in vitro* cultured for 7 days in the presence of Fetal Bovine Serum and in the presence of pericardial fluid at 25%, 50%, 75% and 100%. Representative histograms of four different experiments are shown (A). The expression level of Stem Cell Markers (CD29, CD31, CD44, CD90, CD105) and Swine Leukocyte Antigen Class-I and Class-II (SLA-I and SLA-II) is represented as Normalized Mean Relative Fluorescence Intensity which is calculated by dividing the Mean Fluorescent Intensity (MFI) by its isotype control. Graphic representation of mean±SD for each marker is also provided (n = 4) (B). No significant differences were found between groups when ANOVA test was performed.

Finally, in order to confirm that the phenotype was not affected by the presence of PF, *in vitro* assays were performed using pBM-MSCs co-cultured in the presence of different PFs at different concentrations. No phenotypic differences were observed when different concentrations of PF were used (Fig. 2). These experiments were also conducted for 14 days with similar results to those at 7 days (data not shown).

### Optimized labeling of mesenchymal stem cells for magnetic resonance imaging

**Fig 3:**
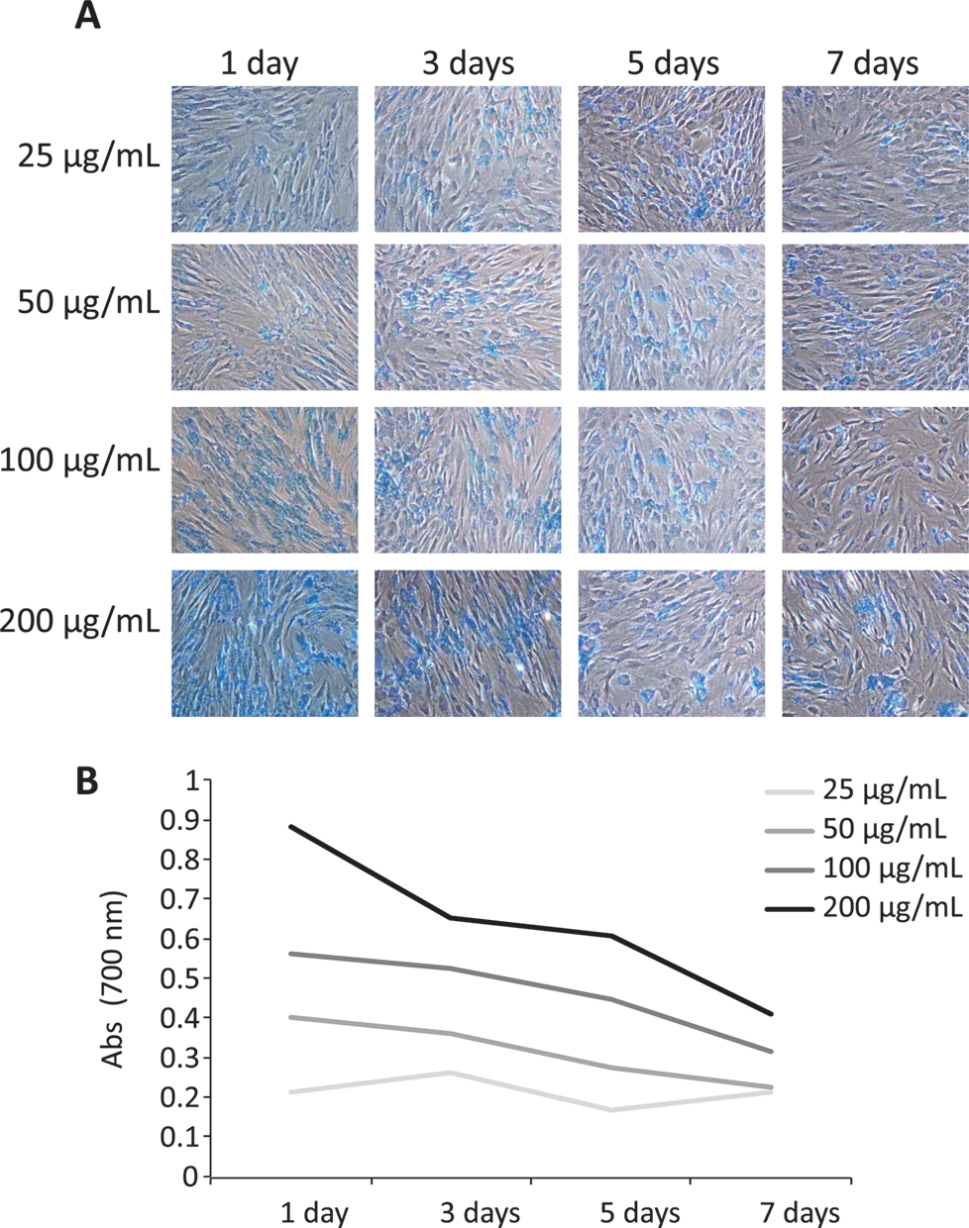
Optimized labeling of pBM-MSCs for MRI. (A) Endorem was incubated with pBM-MSCs at different concentrations (25 μg/ml, 50 μg/ml, 100 μg/ml and 200 μg/ml) Superparamagnetic iron oxide particle were detected by Prussian Blue-staining and observed by optical microscopy. (B) The Prussian Blue staining was spectrophotometrically quantified on pBM-MSCs. The cells were lysed with 0.1% Triton X-100 and the absorbance of the extracts was quantified at 700 nm.

The Endorem is a superparamagnetic particle which can be easily incorporated by endocytosis in MSCs for the *in vivo* cell tracking using MRI. In order to preserve a strong enough T2 signal for *in vivo* experiments preliminary *in vitro* assays were performed. The pBM-MSCs were treated with SPIO at different concentrations and intracellular SPIO was quantified at different time points. Fig. 3A shows the SPIO-labeled cells stained by the Prussian Blue solution. The microscopic images demonstrated that SPIO labeling was proportionally increased to the concentration of SPIO and decreased with time. Moreover, the intracellular SPIO was spectrophotometrically quantified (Fig. 3B) allowing us to conclude that, for an accurate *in vivo* cell tracking, MRI should be limited to 7 days at most.

### Cardiovascular magnetic resonance imaging

The *in vivo* experiments were performed in Large White pigs (n = 4) and a total of 100 x 10^6^ pBM-MSCs were intrapericardially administered via thoracotomy. The *in vivo* tracking of SPIO-labeled cells after intrapericardial administration was monitored using CINE-MRI (S1–S4 Videos) and cardiac-MRI imaging at different time points (S1 and S2 Figs.).

**Fig 4:**
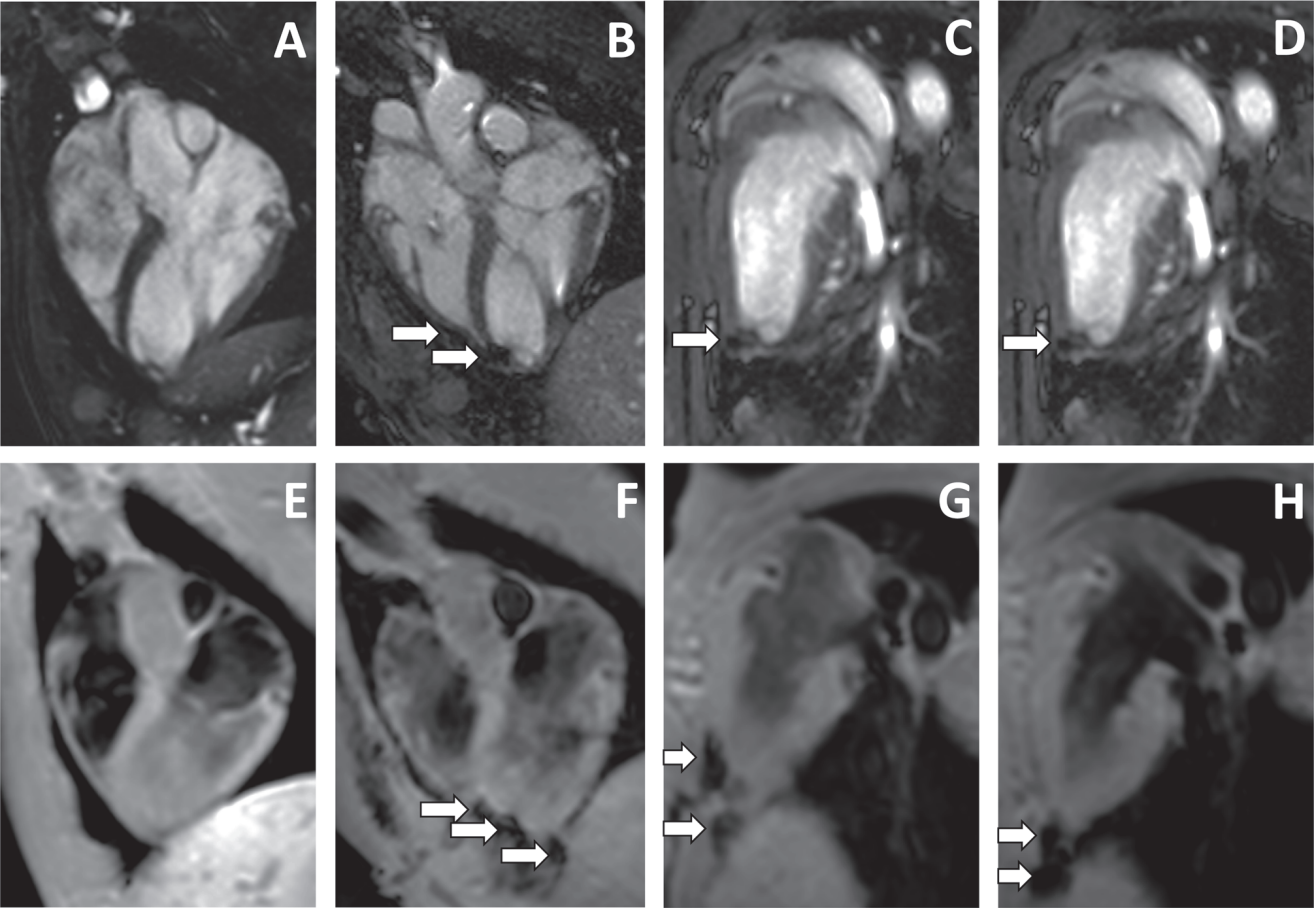
*In vivo* cell tracking of intrapericardially delivered pBM-MSCs by cardiac-MRI in non-infarcted hearts. A total of 100x10^6^ SPIO-labeled pBM-MSCs cells were injected into the pericardial cavity of healthy Large White pigs. The MRI was performed using a 1.5T magnetic resonance technology. Images were acquired in four chamber views (A-D) and using a T2-star gradient echo image (E-H). Images taken at day 3 post injection (B-D, F-H) are represented together with the corresponding control images (A, E) taken before injection. The arrows indicate the location of SPIO-labeled cells.

**Fig 5:**
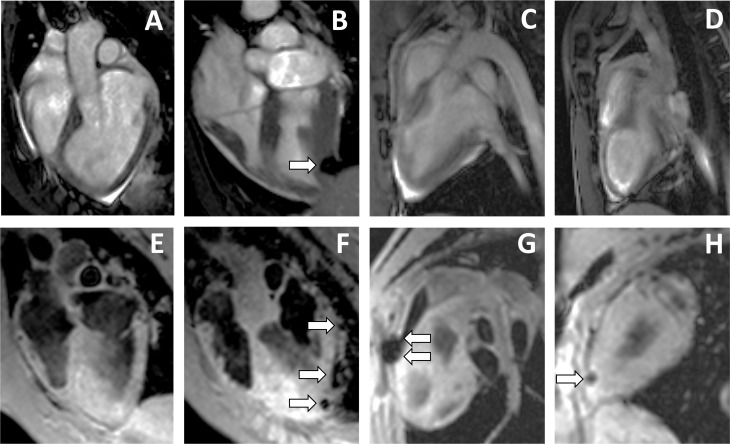
*In vivo* cell tracking of intrapericardially delivered pBM-MSCs by cardiac MRI in infarcted heart. A total of 100x10^6^ SPIO-labeled pBM-MSCs cells were injected into the pericardial cavity. The MRI was performed using 1.5T magnetic resonance technology. Images were acquired in four chambers view (A-D) and using a T2-star gradient echo image (E-H). Images taken at day 3 post injection (B-D, F-H) are represented together with the corresponding control images (A, E) taken before injection and one week after the infarction induction. The arrows indicate the location of SPIO-labeled cells.

The MRI images from non-infarcted hearts clearly demonstrated a preferential distribution of cells in the left ventricle at different time points (Fig. 4). The MRI images from infarcted heart showed that intrapericardial administration of pBM-MSCs rendered a more restricted location of cells in the left ventricle than in non-infarcted heart (Fig. 5).

Finally, it is important to note that, due to the limited sensitivity of MRI, we cannot discard the presence of administered cells in other heart locations. For this, to finely detect transferred cells in the heart, histological studies were performed after euthanasia.

### Macroscopic examination of the heart and histological localization of SPIO and fluorescent-labeled pBM-MSCs

**Fig 6:**
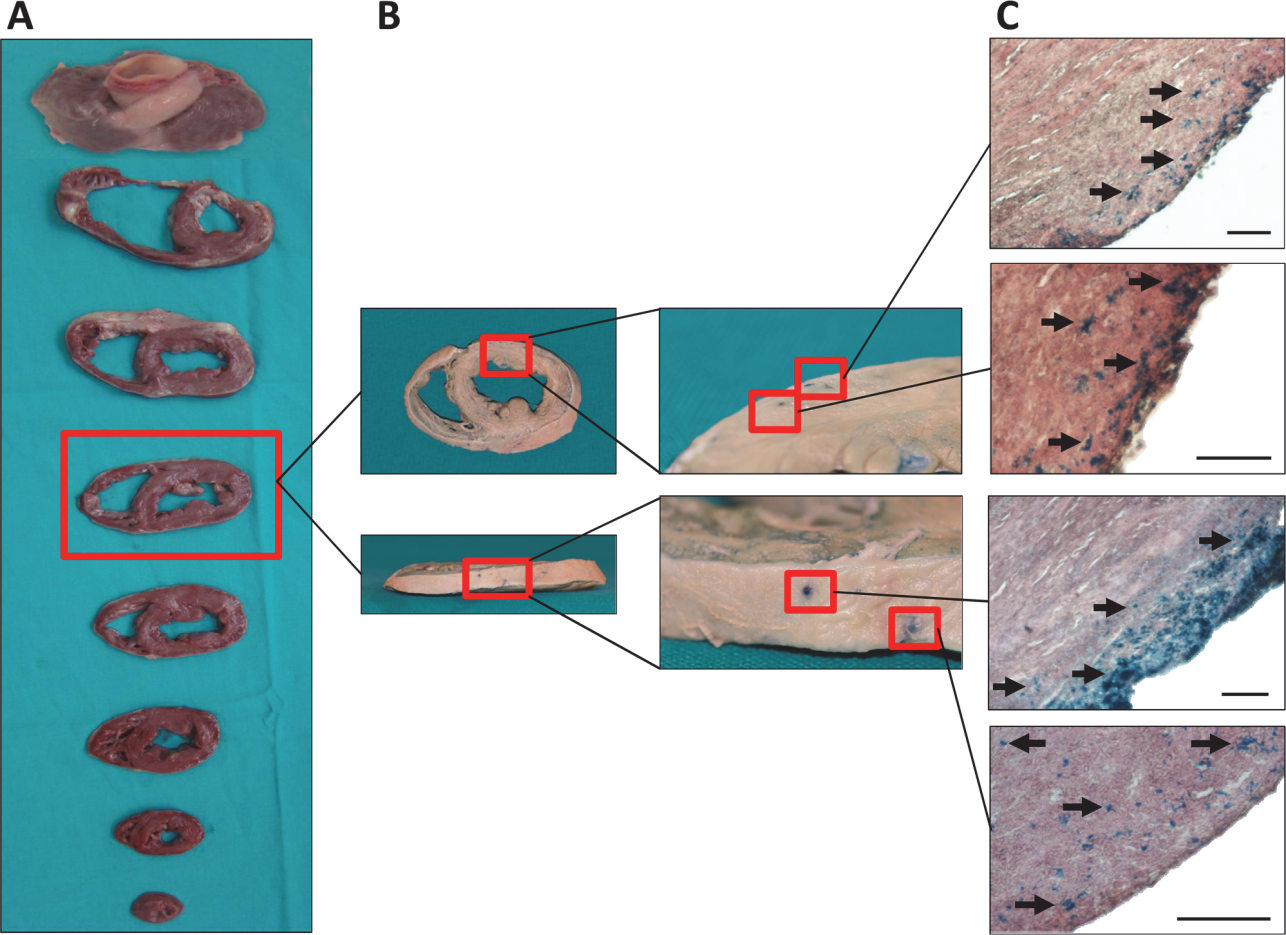
Macroscopic examination and engraftment of SPIO-labeled cells in non-infarcted heart. The SPIO-labeled pBM-MSCs were intrapericardially injected. At 7 days, heart samples were sliced into 1–3 cm short-axis sections and were then photographed (A). The heart slices were fixed in 4% formaldehyde. For the detection of SPIO–labeled cells, tissue sections were incubated with 8% hydrochloric acid and 4% potassium ferrocyanide and then with eosin. The blue color indicates the presence of SPIO within the tissue (B). Finally, for histological examination, tissue sections were paraffin-embedded and Prussian-blue/eosin staining demonstrated a preferential distribution of SPIO–labeled cells in the left ventricular myocardium (C). Scale bar: 100μm. Black arrows indicate the presence of SPIO-labeled cells evidenced by the potassium ferrocyanide staining.

**Fig 7:**
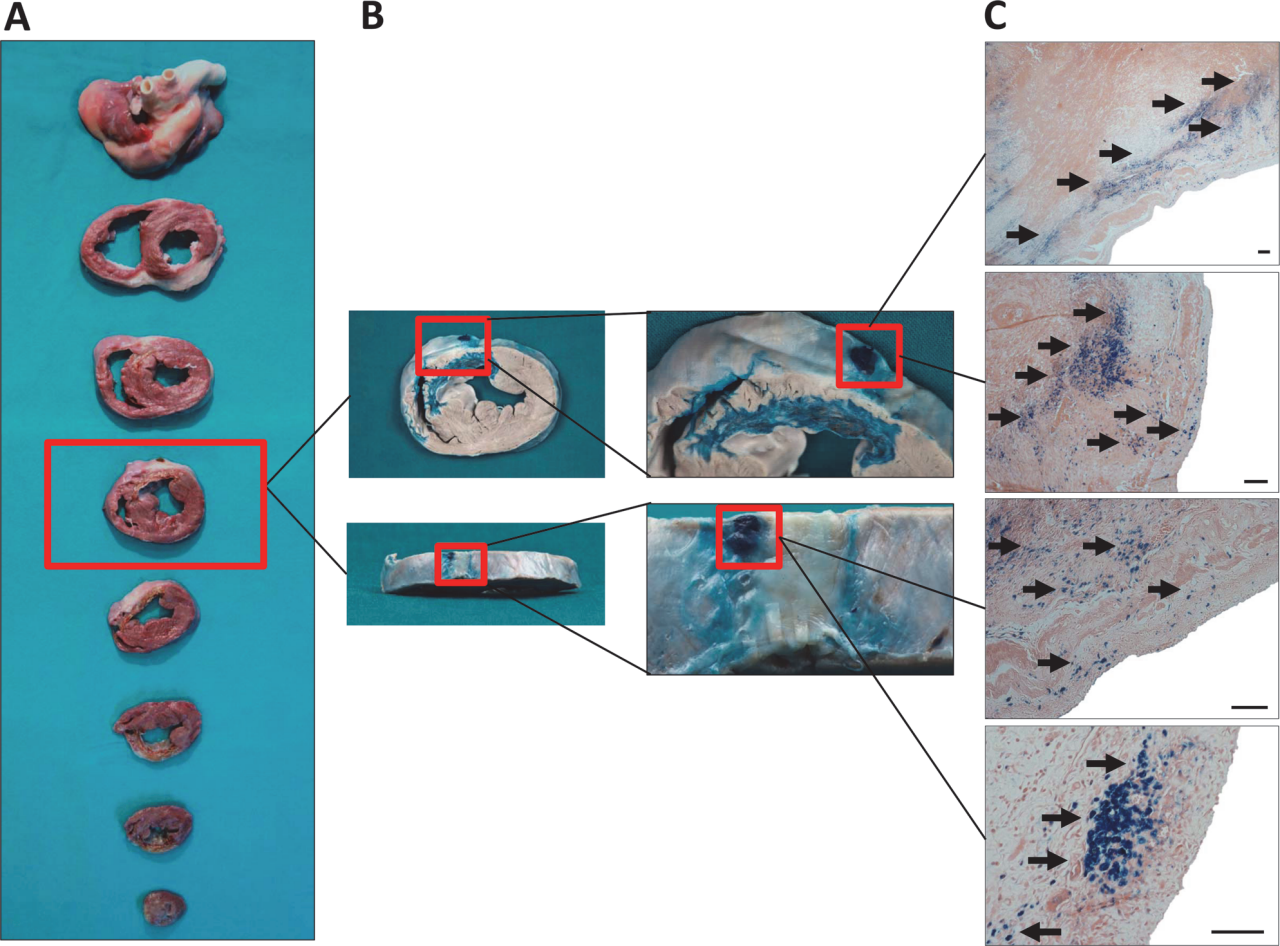
Macroscopic examination and engraftment of SPIO-labeled cells in an infarcted heart. The SPIO-labeled pBM-MSCs were intrapericardially injected one week after infarction induction. At 7 days, heart samples were sliced into 1–3 cm short-axis sections and were then photographed (A). The heart slices were fixed in 4% formaldehyde. For the detection of SPIO–labeled cells, tissue sections were incubated with 8% hydrochloric acid and 4% potassium ferrocyanide and then with eosin. The blue color indicates the presence of SPIO within the tissue (B). Finally, for histological examination, tissue sections were paraffin-embedded and Prussian-blue/eosin staining demonstrated a preferential distribution of SPIO–labeled cells in the left ventricular myocardium (C). Scale bar: 100μm. Black arrows indicate the presence of SPIO-labeled cells evidenced by the potassium ferrocyanide staining.

Seven days after intrapericardial administration, the animals were euthanized and thoracic cavity was opened. A visual examination showed a normal conformation of tissues, with few pericardial adherences to thoracic wall due to the surgical intervention. The PF was recovered before collecting the samples for histological and molecular analysis. The volume and macroscopic aspect of the fluid were normal indicating an absence of pericarditis. A biochemical analysis revealed not significant changes before and after the surgery (data not shown). Finally, the pericardium and the heart were removed and rinsed in saline serum. A detailed macroscopic evaluation was also performed but any important alteration was found on non-infarcted or infarcted hearts (Figs. 6A and 7A).

For histological and molecular studies, the hearts were sliced into several sections. These sections were fixed and submerged in a Prussian Blue solution and heart slices showed positive macroscopic reactions in the epicardium and endocardium layers of the heart (Figs. 6B and 7B). The histological comparison between non-infarcted and infarcted heart corroborates the MRI findings. A preferential location of cells was observed in the left ventricle with a more diffuse distribution of cells in non-infarcted myocardium. In contrast, the cell distribution was more restricted to a small area in the infarcted myocardium.

In order to confirm the migratory behavior of transferred cells, tissue samples were collected and fixed for histological examination. For the visualization of SPIO-labeled cells, paraffin sections were stained with Prussian Blue solution and then counterstained with eosin. As shown in Figs. 6C and 7C, SPIO-labeled cells could be found at day 7 in the inner tissue after intrapericardial administration. Similar results were obtained using fluorescent-labeled cells detected by fluorescence microscopy (S3 Fig.). Moreover, histological evaluations with Masson Trichrome and Toluidine blue showed non-adverse effects in animal tissues (S4 Fig.).

### Y chromosome detection into the female recipients

**Fig 8:**
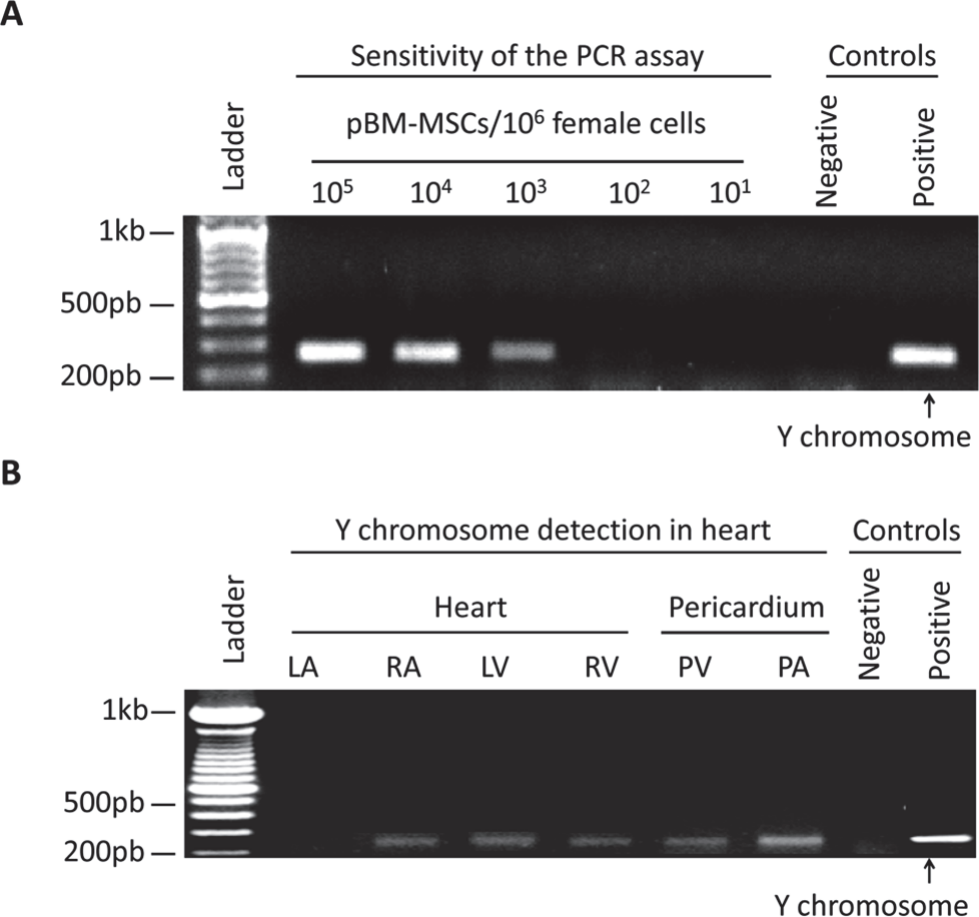
Y chromosome detection of intrapericardially delivered pBM-MSCs. The pBM-MSCs from a male donor were detected in the heart by Y chromosome amplification. (A) In order to determine the sensitivity of PCR amplification, pBM-MSCs from a male donor were mixed with 10^6^ pBM-MSCs from a female donor at the indicated ratios. Genomic DNA was extracted and subjected to PCR amplification using Y chromosome specific primers. The PCR allowed the detection of male cells with a sensitivity of 100–1000 cells per 10^6^ female cells. (B) Female pigs were intrapericardially injected with male-derived pBM-MSCs. At day 7, the animals were euthanized and heart samples were collected for PCR analysis. As negative and positive controls, the genomic DNA from female and male pBM-MSCs cells were amplified. LA = left atrium, LV = left ventricle, RA = right atrium, RV = right ventricle, PV = pericardium on the right and left ventricles, PA = pericardium on the right and left atrium.

In order to determine the detection limit of the technique, preliminary experiments were performed. Samples with 10^1^, 10^2^, 10^3^, 10^4^ and 10^5^ male cells were mixed together with one million female cells and amplified by PCR. This technique showed a detection limit of 100–1000 male cells per 10^6^ female cells (Fig. 8A). For the detection of transferred cells, the DNA from different tissues was isolated and Y chromosome amplified by PCR. As shown in Fig. 8B, the pBM-MSCs could be detected in samples from left ventricle, right atrium and right ventricle as well as in the pericardial sections surrounding the ventricles and atria. Taking into consideration that false SPIO staining from free particles could occur, the Y-chromosome amplification clearly confirmed the persistence of injected cells in the heart and pericardium after 7 days.

## Discussion

The safety and efficacy of intrapericardial therapies have been demonstrated in multiple studies [27,28] and large animal models such as pigs have been widely used for the evaluation of different compounds [24,25]. This work aimed to analyze the suitability of PF as a vehicle for MSCs administration together with the biodistribution of intrapericardially administered MSCs in a clinically relevant animal model. The swine present many similarities to the human heart and there is an increasing amount of preclinical works that can help interpreting and putting the results in context [29].

At the present, bibliography shows several evidences for the successful integration of stem cells in the heart [30], being widely accepted that the administration route is one of the major factors influencing their therapeutic effect [14]. For stem cell-based therapies, the viability, survival and migratory behavior of transferred cells (especially in myocardial infarction and stroke), should be guaranteed in the target tissue [31]. In this sense, our first sets of experiments were conducted to evaluate the PF as a vehicle for stem cell delivery.

We firstly aimed to compare the biochemical profile between PF and plasma (an innocuous medium for cells growing and surviving). Our results demonstrated significant differences in the biochemical composition which is normal considering that PF is an ultrafiltrate of plasma. Moreover, the *in vitro* experiments demonstrated that PF provided an optimal viability, proliferation and long term survival of MSCs. To our knowledge, this is the first report where it has been experimentally demonstrated that PF is an optimal vehicle for MSCs.

According to these *in vitro* observations, we hypothesized that the intrapericardial administration of MSCs may retain the cells in close proximity to the injury preserving its bioactivity. Based on this hypothesis, our *in vivo* experiments were conducted in a clinically relevant animal model. In these experiments, animals received a higher dose of MSCs but no inflammation, pericardial effusion or adverse adhesions were noted. Considering that safety is a major issue in cardiovascular stem cell therapy, our findings suggest that intrapericardial delivery is a safe route for MSCs transplantation. These results are in agreement with previous reports where the combined usage of MSCs and biomaterials were safely administered by this route [32,33].

The *in vivo* cell tracking by MRI clearly showed that administered cells were preferentially located surrounding the left ventricle. These results are relevant because myocardial infarction frequently involves the left ventricle [34], so this route may initially provide an optimal cell location and retention rate in the region of interest. Interestingly, previous results from our group, using intramyocardial delivery in the same animal model under similar conditions [35], showed that cell distribution and retention is comparable to that by intrapericardial administration.

It is important to note that, *in vivo* cell tracking of SPIO-labeled cells still remains controversial because free SPIO particles may cause false positives in the resonance that leads to an inaccurate analysis [36]. These false positives could be observed as a consequence of SPIO exocytosis from live cells [37]. Moreover, another commonly encountered problem is that, if cells die, magnetic particles will still be imaged by MRI and may be internalized by other cells [38]. In this work, our *in vitro* results suggest that, in preclinical settings a short-term follow-up could be feasible for at least 7 days. Moreover, in order to exclude the possibility that the totality of SPIO from MSCs could be internalized by resident phagocytic cells (i.e. macrophages), PCR analysis and histological studies were performed. The Y-chromosome amplification together with fluorescent microscopy images using fluorescent-labeled MSCs clearly demonstrated an engraftment of MSCs in the myocardium. However, although PCR amplification and fluorescent images demonstrated the presence of MSCs in the heart, we should also assume that some of the SPIO-positive cells observed in the tissue samples would also correspond to phagocytic cells [39,40].

In this work, there is a noteworthy limitation concerning to the *in vivo* efficacy of administered cells under acute myocardial infarct conditions. In this sense, *in vivo* studies are currently being conducted in a porcine model of myocardial infarct to evaluate the therapeutic effect of administered cells as well as the time-course and physiological conditions where the cells may display their beneficial effects (manuscript in preparation).

Regarding to the immunological aspects, it has been hypothesized that, the introduction of exogenous stem cells may be hampered by immune rejection [41]. In the case of intrapericardial delivery, the immune response against transferred cells could be mediated by activated lymphocytes in the pericardial sac. In fact, the presence of such lymphocytes has been previously described in patients with different forms of heart disease [42]. For this, although MSCs possess a potent immunosuppressive function [43] we cannot discard a hypothetical immune response against transferred allogeneic MSCs and this aspect is currently under study in our laboratory.

Respecting to the hypothetical inefficient homing commonly linked to the administration of exogenous stem cells, our histological and PCR analysis have clearly confirmed the presence of transferred cells in different locations of the heart demonstrating that MSCs efficiently migrate from PF into the heart. Additionally we should also consider that, lymphatic drainage of the PF could be considered an “open door” for MSCs that may result in a systemic distribution of intrapericardially administered cells.

In conclusion, our results demonstrated that PF is an optimal vehicle for MSCs and intrapericardial administration is an optimal route for MSCs transplantation. This route has the great advantage of transferring relatively large amounts of cells avoiding the inherent risk of cell embolism linked to intracoronary administration. Moreover, in contrast to the local intramyocardial administration of cells where the underperfused myocardium makes an unfavored environment for cell survival, the intrapericardial delivery may provide an optimal environment for maintaining cell viability.

## Supporting Information

S1 Fig Time-course of *in vivo* cell tracking of intrapericardially delivered pBM-MSCs by cardiac-MRI in non-infarcted heart. SPIO magnetic signal was detected by resonance for up to a week after injection. The MRI was performed using a 1.5T magnetic resonance technology. Images were acquired in four chamber views (A-D) and using a T2-star gradient echo image (E-H). Representative images of the MRI performed before the injection (A,E), after 3 days (B,F), 5 days (C,G) and 7 days post-injection (D, H) are shown. The arrows indicate the location of SPIO signal.
(TIF)

S2 Fig Time-course of *in vivo* cell tracking of intrapericardially delivered pBM-MSCs by cardiac-MRI in an infarcted heart. SPIO magnetic signal was detected by resonance for up to a week after injection. The MRI was performed using a 1.5T magnetic resonance technology. Images were acquired in four chamber views (A-D) and using a T2-star gradient echo image (E-H). Representative images of the MRI performed before the injection (A,E), after 3 days (B,F), 5 days (C,G) and 7 days post-injection (D, H) are shown. The arrows indicate the location of SPIO signal.
(TIF)

S3 Fig Engraftment of fluorescent-labeled pBM-MSCs in the heart. For the detection of Vybrant-labeled cells, tissue sections were fixed, paraffin-embedded and stained using the Masson’s Trichrome Staining Protocol. The engraftment of Vybrant-labeled cells was visualized under fluorescent microscope. The A, B and C images correspond to an optical microscope image, fluorescent microscope image and merged them respectively. Scale bar: 100 μm.
(TIF)

S4 Fig Histological section in the left ventricle from animals sacrificed at day 7 post-administration. Tissue sections were fixed, paraffine-embedded and stained using Toluidine-Blue (A, B) or the Masson's Trichrome staining protocol (C, D). The stainings were visualized at 4X (left column) and 10X (right column) objective magnification. Scale bars: 500 μm and 100μm for 4X and 10X respectively.
(TIF)

S1 Video Four chambers cine loop (T2_BTFE_BH) of non-infarcted heart at day 3 post-injection. The MRI was performed using a 1.5T magnetic resonance technology. Images were acquired in four chamber views. SPIO nanoparticles signal can be observed in the region corresponding to the apex and left ventricle. White intermittent arrows indicate the presence of SPIO-labeled cells.
(3GP)

S2 Video Long axis cine loop (T2_BTFE_BH) of non-infarcted heart at day 3 post-injection. The MRI was performed using a 1.5T magnetic resonance technology. SPIO nanoparticles signal can be observed in the region corresponding to the apex and left ventricle. White intermittent arrows indicate the presence of SPIO-labeled cells.
(3GP)

S3 Video Four chambers cine loop (T2_BTFE_BH) of an infarcted heart at day 3 post-injection. The MRI was performed using a 1.5T magnetic resonance technology. Images were acquired in four chamber views. SPIO nanoparticles signal can be observed in the region corresponding to the apex and left ventricle. White intermittent arrows indicate the presence of SPIO-labeled cells.
(3GP)

S4 Video Long axis cine loop (T2_BTFE_BH) of an infarcted heart at day 3 post-injection. The MRI was performed using a 1.5T magnetic resonance technology. SPIO nanoparticles signal can be observed in the region corresponding to the apex and left ventricle. White intermittent arrows indicate the presence of SPIO-labeled cells.
(3GP)

## References

[1] Williams AR, Hare JM. Mesenchymal stem cells: Biology, patho-physiology, translational findings, and therapeutic implications for cardiac disease. Circ Res. 2011; 109(8):923–940. 10.1161/CIRCRESAHA.111.243147 21960725 PMC3604746

[2] Sheng CC, Zhou L, Hao J. Current stem cell delivery methods for myocardial repair. Biomed Res Int. 2013; 2013:547902 Available: http://www.hindawi.com/journals/bmri/2013/547902. 10.1155/2013/547902 23509740 PMC3591183

[3] Mazo M, Gavira JJ, Pelacho B, Prosper F. Adipose-derived stem cells for myocardial infarction. J Cardiovasc Transl Res. 2011; 4(2):145–153. 10.1007/s12265-010-9246-y 21116883

[4] Plant AL, Parker GC. Translating stem cell research from the bench to the clinic: a need for better quality data. Stem Cells Dev. 2013; 22(18):2457–2458. 10.1089/scd.2013.0188 23597110

[5] Lalu MM, McIntyre L, Pugliese C, Fergusson D, Winston BW, Marshall JC, et al Safety of cell therapy with mesenchymal stromal cells (SafeCell): a systematic review and meta-analysis of clinical trials. PLoS One. 2012; 7(10):e47559 Available: http://www.plosone.org/article/info%3Adoi%2F10.1371%2Fjournal.pone.0047559. 10.1371/journal.pone.0047559 23133515 PMC3485008

[6] Trivedi P, Tray N, Nguyen T, Nigam N, Gallicano GI. Mesenchymal stem cell therapy for treatment of cardiovascular disease: helping people sooner or later. Stem Cells Dev. 2010; 19(7):1109–1120. 10.1089/scd.2009.0465 20092388

[7] Hoogduijn MJ, Roemeling-van RM, Korevaar SS, Engela AU, Weimar W, Baan CC. Immunological aspects of allogeneic and autologous mesenchymal stem cell therapies. Hum Gene Ther. 2011; 22(12):1587–1591. 10.1089/hum.2011.039 21732766

[8] Gebler A, Zabel O, Seliger B. The immunomodulatory capacity of mesenchymal stem cells. Trends Mol Med. 2011; 18(2): 128–134. 10.1016/j.molmed.2011.10.004 22118960

[9] Huang NF, Lam A, Fang Q, Sievers RE, Li S, Lee RJ. Bone marrow-derived mesenchymal stem cells in fibrin augment angiogenesis in the chronically infarcted myocardium. Regen Med. 2009; 4(4):527–538. 10.2217/rme.09.32 19580402 PMC2778008

[10] Sanganalmath SK, Bolli R. Cell therapy for heart failure: a comprehensive overview of experimental and clinical studies, current challenges, and future directions. Circ Res. 2013; 113(6):810–834. 10.1161/CIRCRESAHA.113.300219 23989721 PMC3892665

[11] Freyman T, Polin G, Osman H, Crary J, Lu M, Cheng L, et al A quantitative, randomized study evaluating three methods of mesenchymal stem cell delivery following myocardial infarction. Eur Heart J. 2006; 27(9):1114–1122. 16510464 10.1093/eurheartj/ehi818

[12] van RJ, Rodrigo SF, Schalij MJ, Beeres SL, Bax JJ, Atsma DE. Bone marrow cell injection for chronic myocardial ischemia: the past and the future. J Cardiovasc Transl Res. 2011; 4(2):182–191. 10.1007/s12265-010-9249-8 21213093 PMC3047688

[13] Dib N, Khawaja H, Varner S, McCarthy M, Campbell A. Cell therapy for cardiovascular disease: a comparison of methods of delivery. J Cardiovasc Transl Res. 2011; 4(2):177–181. 10.1007/s12265-010-9253-z 21181320 PMC3047684

[14] Fukushima S, Sawa Y, Suzuki K. Choice of cell-delivery route for successful cell transplantation therapy for the heart. Future Cardiol. 2013; 9(2):215–227. 10.2217/fca.12.85 23463974

[15] Fukushima S, Varela-Carver A, Coppen SR, Yamahara K, Felkin LE, Lee J, et al Direct intramyocardial but not intracoronary injection of bone marrow cells induces ventricular arrhythmias in a rat chronic ischemic heart failure model. Circulation. 2007; 115(17): 2254–2261. 17438152 10.1161/CIRCULATIONAHA.106.662577

[16] Morrison SJ, Spradling AC. Stem cells and niches: mechanisms that promote stem cell maintenance throughout life. Cell. 2008; 132(4): 598–611. 10.1016/j.cell.2008.01.038 18295578 PMC4505728

[17] Barbash IM, Chouraqui P, Baron J, Feinberg MS, Etzion S, Tessone A, et al Systemic delivery of bone marrow-derived mesenchymal stem cells to the infarcted myocardium: feasibility, cell migration, and body distribution. Circulation. 2003; 108(7): 863–868. 12900340 10.1161/01.CIR.0000084828.50310.6A

[18] Grieve SM, Bhindi R, Seow J, Doyle A, Turner AJ, Tomka J, et al Microvascular Obstruction by Intracoronary Delivery of Mesenchymal Stem Cells and Quantification of Resulting Myocardial Infarction by Cardiac Magnetic Resonance. Circ Heart Fail. 2010; 3(3): e5–e6. 10.1161/CIRCHEARTFAILURE.109.931360 20484192

[19] Vulliet PR, Greeley M, Halloran SM, MacDonald KA, Kittleson MD. Intra-coronary arterial injection of mesenchymal stromal cells and microinfarction in dogs. Lancet. 2004; 363(9411): 783–784. 15016490 10.1016/S0140-6736(04)15695-X

[20] Sun F, Sanchez FM, Fernandez-Portales J, Crisostomo V, Diaz-Guemes I, Baez-Diaz C, et al Chronic intrapericardial catheterization for repeated drug delivery: technical feasibility study in the Gottingen minipig. J Invasive Cardiol. 2012; 24(5):210–214. 22562914

[21] Sun F, Sanchez FM, Crisostomo V, Luis L, Uson J, Maynar M. Subxiphoid access to normal pericardium with micropuncture set: technical feasibility study in pigs. Radiology. 2006; 238(2):719–724. 16371586 10.1148/radiol.2382042182

[22] Laham RJ, Rezaee M, Post M, Xu X, Sellke FW. Intrapericardial administration of basic fibroblast growth factor: myocardial and tissue distribution and comparison with intracoronary and intravenous administration. Catheter Cardiovasc Interv. 2003; 58(3):375–381. 12594706 10.1002/ccd.10378

[23] Fei L, Baron AD, Henry DP, Zipes DP. Intrapericardial delivery of L-arginine reduces the increased severity of ventricular arrhythmias during sympathetic stimulation in dogs with acute coronary occlusion: nitric oxide modulates sympathetic effects on ventricular electrophysiological properties. Circulation. 1997; 96(11):4044–4049. 9403630 10.1161/01.cir.96.11.4044

[24] Xiao YF, Sigg DC, Ujhelyi MR, Wilhelm JJ, Richardson ES, Iaizzo PA. Pericardial delivery of omega-3 fatty acid: a novel approach to reducing myocardial infarct sizes and arrhythmias. Am J Physiol Heart Circ Physiol. 2008; 294(5):H2212–H2218. 10.1152/ajpheart.91502.2007 18326793

[25] Laham RJ, Rezaee M, Post M, Novicki D, Sellke FW, Pearlman JD, et al Intrapericardial delivery of fibroblast growth factor-2 induces neovascularization in a porcine model of chronic myocardial ischemia. J Pharmacol Exp Ther. 2000; 292(2):795–802. 10640320

[26] Rupp H, Rupp TP, Alter P, Jung N, Pankuweit S, Maisch B. Intrapericardial procedures for cardiac regeneration by stem cells: need for minimal invasive access (AttachLifter) to the normal pericardial cavity. Herz. 2010; 35(7):458–465. 10.1007/s00059-010-3382-7 20941468

[27] Kornowski R, Fuchs S, Leon MB, Epstein SE. Delivery strategies to achieve therapeutic myocardial angiogenesis. Circulation. 2010; 101(4):454–458. 10.1161/01.cir.101.4.454 10653839

[28] Maisch B, Pankuweit S. Current treatment options in (peri)myocarditis and inflammatory cardiomyopathy. Herz. 2012; 37(6):644–656. 22996288 10.1007/s00059-012-3679-9

[29] Crisostomo V, Maestre J, Maynar M, Sun F, Baez-Diaz C, Uson J, et al. Development of a closed chest model of chronic myocardial infarction in Swine: magnetic resonance imaging and pathological evaluation. ISRN Cardiol. 2013:781762. Available: http://www.hindawi.com/journals/isrn.cardiology/2013/781762. 10.1155/2013/781762 PMC3825272 24282645

[30] Sharma R, Raghubir R. Stem cell therapy: a hope for dying hearts. Stem Cells Dev. 2007; 16(4):517–536. 17784827 10.1089/scd.2006.0070

[31] Hyun JS, Tran MC, Wong VW, Chung MT, Lo DD, Montoro DT, et al Enhancing stem cell survival in vivo for tissue repair. Biotechnol Adv. 2013; 31(5):736–743. 10.1016/j.biotechadv.2012.11.003 23153460

[32] Ladage D, Turnbull IC, Ishikawa K, Takewa Y, Rapti K, Morel C, et al Delivery of gelfoam-enabled cells and vectors into the pericardial space using a percutaneous approach in a porcine model. Gene Ther. 2011; 18(10):979–985. 10.1038/gt.2011.52 21512506 PMC3651891

[33] Yang Y, Dreessen de GP, Sun J, Glogowski M, Gussakovsky E, Kupriyanov V. MRI studies of cryoinjury infarction in pig hearts: ii. Effects of intrapericardial delivery of adipose-derived stem cells (ADSC) embedded in agarose gel. NMR Biomed. 2012; 25(2):227–235. 10.1002/nbm.1735 21774011

[34] Ruiz-Esparza GU, Flores-Arredondo JH, Segura-Ibarra V, Torre-Amione G, Ferrari M, Blanco E, et al The physiology of cardiovascular disease and innovative liposomal platforms for therapy. Int J Nanomedicine. 2013; 8:629–640. 10.2147/IJN.S30599 23413209 PMC3572823

[35] Gomez-Mauricio RG, Acarregui A, Sánchez-Margallo FM, Crisóstomo V, Gallo I, Hernandez RM, et al A preliminary approach to the repair of myocardial infarction using adipose tissue-derived stem cells encapsulated in magnetic resonance-labelled alginate microspheres in a porcine model. Eur J Pharm Biopharm. 2013; 84(1): 29–39. 10.1016/j.ejpb.2012.11.028 23266493

[36] Jasmin, Torres AL, Jelicks L, de Carvalho AC, Spray DC, Mendez-Otero R. Labeling stem cells with superparamagnetic iron oxide nanoparticles: analysis of the labeling efficacy by microscopy and magnetic resonance imaging. Methods Mol Biol. 2012; 906:239–52. 10.1007/978-1-61779-953-2_18 22791437 PMC3682662

[37] Cromer Berman SM, Kshitiz, Wang CJ, Orukari I, Levchenko A, Bulte JWM, et al Cell Motility of Neural Stem Cells is Reduced after SPIO-Labeling, which is Mitigated after Exocytosis. Magn Reson Med. 2013; 69(1):255–262. 10.1002/mrm.24216 22374813 PMC3366052

[38] Bull E, Madani SY, Sheth R, Seifalian A, Green M, Seifalian AM. Stem cell tracking using iron oxide nanoparticles. Int J Nanomedicine. 2014; 9:1641–1653. 10.2147/IJN.S48979 24729700 PMC3976208

[39] Amsalem Y, Mardor Y, Feinberg MS, Landa N, Miller L, Daniels D, et al Iron-oxide labeling and outcome of transplanted mesenchymal stem cells in the infarcted myocardium. Circulation. 2007; 116(11 Suppl):I38–I45. 17846324 10.1161/CIRCULATIONAHA.106.680231

[40] Ittrich H, Peldschus K, Raabe N, Kaul M, Adam G. Superparamagnetic iron oxide nanoparticles in biomedicine: applications and developments in diagnostics and therapy. Rofo. 2013; 185(12):1149–1166. 10.1055/s-0033-1335438 24008761

[41] Smart N, Riley PR. The stem cell movement. Circ Res. 2008; 102(10):1155–1168. 10.1161/CIRCRESAHA.108.175158 18497316

[42] Riemann D, Wollert HG, Menschikowski J, Mittenzwei S, Langner J. Immunophenotype of lymphocytes in pericardial fluid from patients with different forms of heart disease. Int Arch Allergy Immunol. 1994; 104(1):48–56. 7950405 10.1159/000236708

[43] Casado JG, Tarazona R, Sanchez-Margallo FM. NK and MSCs crosstalk: the sense of immunomodulation and their sensitivity. Stem Cell Rev. 2013; 9(2):184–189. 10.1007/s12015-013-9430-y 23397451

