# Supplementary figures and images for: Intrapericardial Administration of Mesenchymal Stem Cells in a Large Animal Model: A Bio-Distribution Analysis
Source: PLoS One. 2015 Mar 27;10(3):e0122377. doi: 10.1371/journal.pone.0122377 (PMC4376786; doi:10.1371/journal.pone.0122377)

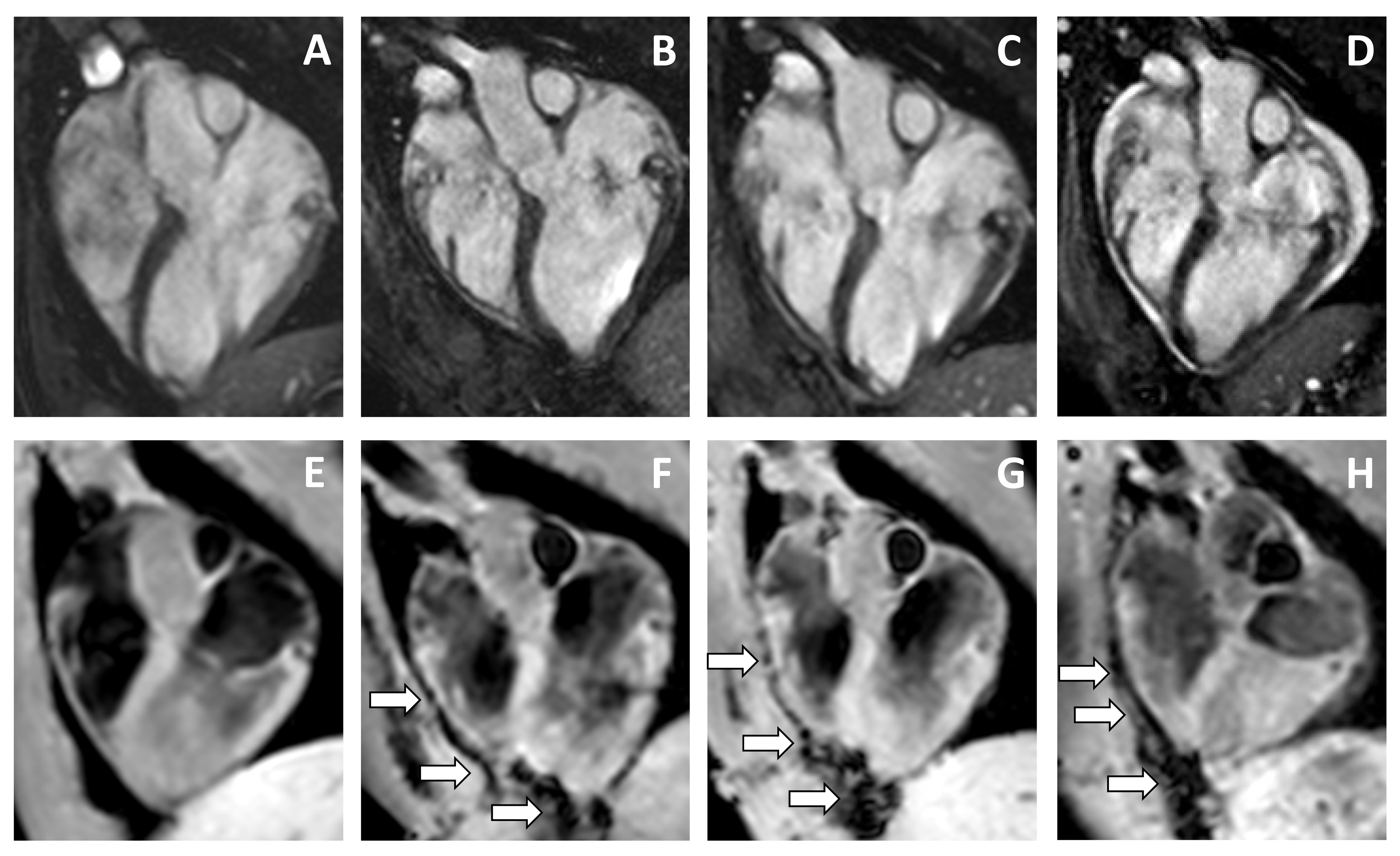

Supplement: S1 Fig — SPIO magnetic signal was detected by resonance for up to a week after injection. The MRI was performed using a 1.5T magnetic resonance technology. Images were acquired in four chamber views (A-D) and using a T2-star gradient echo image (E-H). Representative images of the MRI performed before the injection (A,E), after 3 days (B,F), 5 days (C,G) and 7 days post-injection (D, H) are shown. The arrows indicate the location of SPIO signal. (TIF) [file pone.0122377.s001.tif]

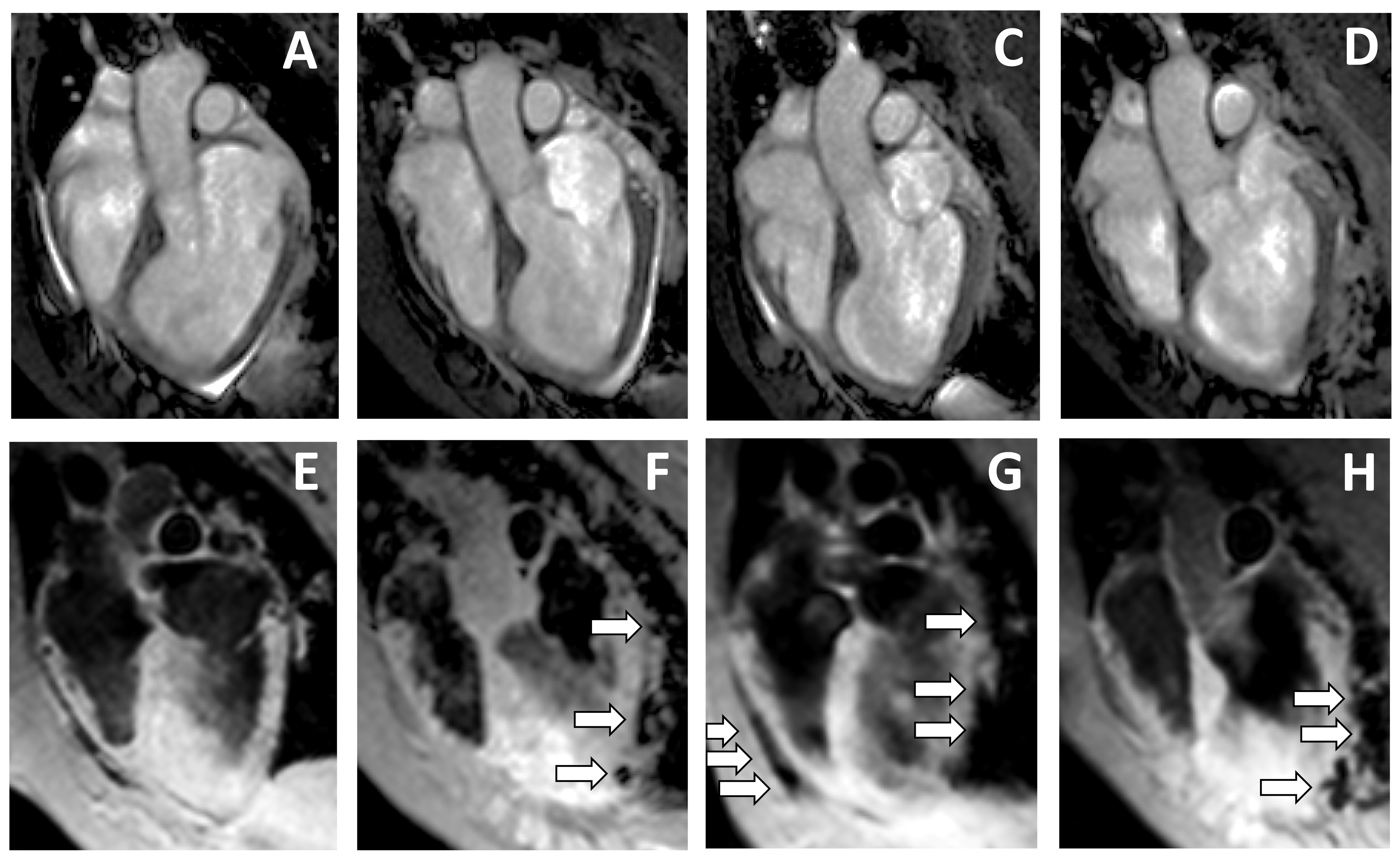

Supplement: S2 Fig — SPIO magnetic signal was detected by resonance for up to a week after injection. The MRI was performed using a 1.5T magnetic resonance technology. Images were acquired in four chamber views (A-D) and using a T2-star gradient echo image (E-H). Representative images of the MRI performed before the injection (A,E), after 3 days (B,F), 5 days (C,G) and 7 days post-injection (D, H) are shown. The arrows indicate the location of SPIO signal. (TIF) [file pone.0122377.s002.tif]

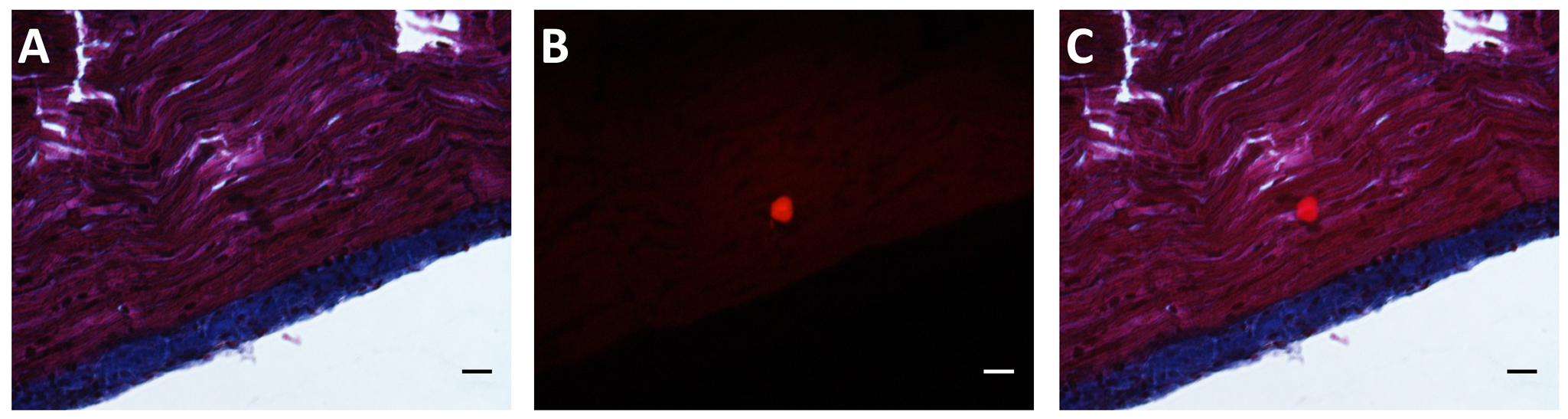

Supplement: S3 Fig — For the detection of Vybrant-labeled cells, tissue sections were fixed, paraffin-embedded and stained using the Masson’s Trichrome Staining Protocol. The engraftment of Vybrant-labeled cells was visualized under fluorescent microscope. The A, B and C images correspond to an optical microscope image, fluorescent microscope image and merged them respectively. Scale bar: 100 μm. (TIF) [file pone.0122377.s003.tif]

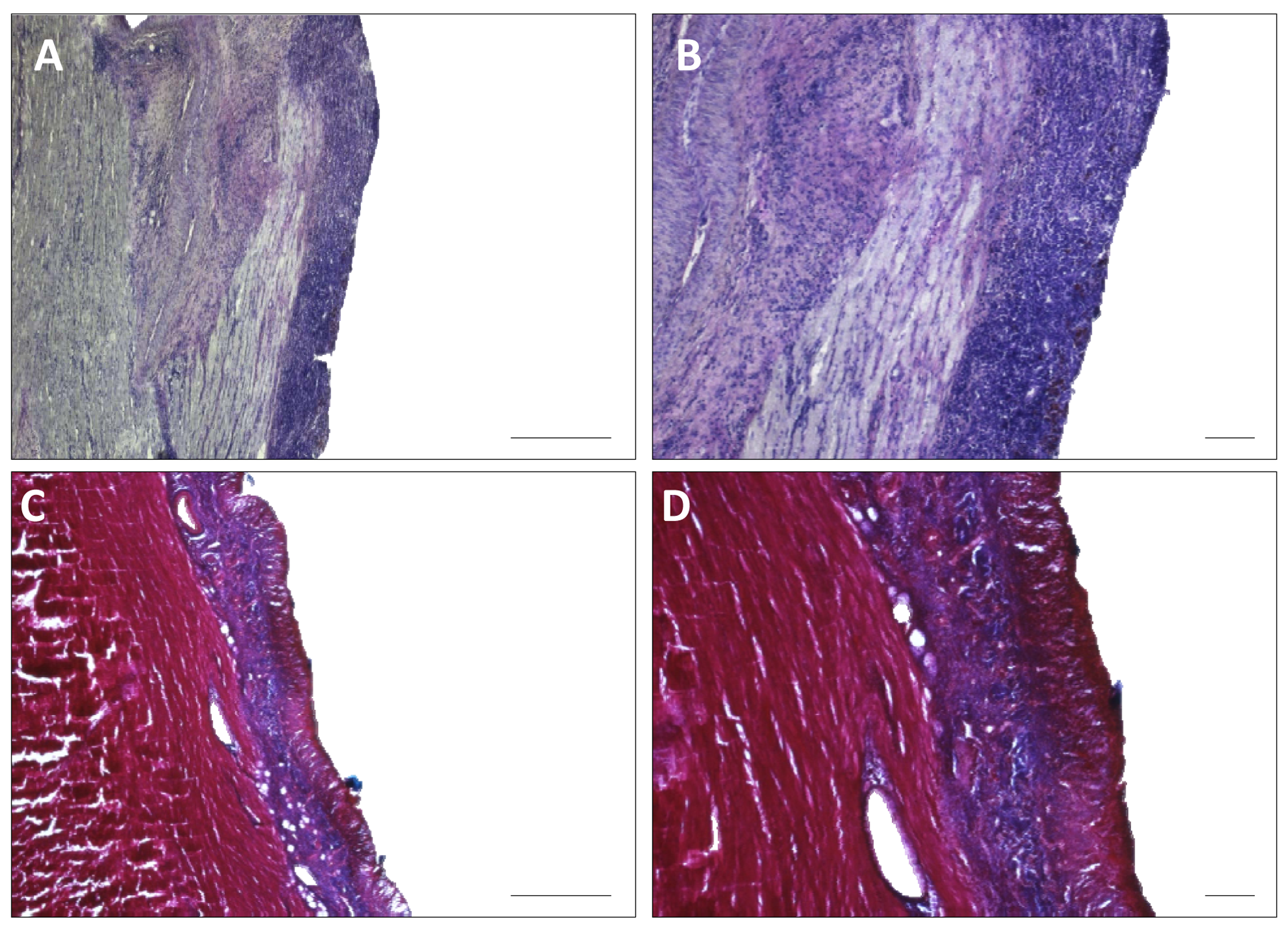

Supplement: S4 Fig — Tissue sections were fixed, paraffine-embedded and stained using Toluidine-Blue (A, B) or the Masson's Trichrome staining protocol (C, D). The stainings were visualized at 4X (left column) and 10X (right column) objective magnification. Scale bars: 500 μm and 100μm for 4X and 10X respectively. (TIF) [file pone.0122377.s004.tif]
